# Significance of semaphorin-3A and MMP-14 protein expression in non-small cell lung cancer

**DOI:** 10.3892/ol.2014.1920

**Published:** 2014-02-27

**Authors:** HAIYING ZHOU, AIPING WU, WEI FU, ZHENG LV, ZHIYONG ZHANG

**Affiliations:** 1 Department of Thoracic Surgery, Tianjin Chest Hospital, Tianjin Medical University, Tianjin 300070, P.R. China; 2 Department of Thoracic Surgery, Tangshan Worker Hospital, Hebei Medical University, Tangshan, Hebei 063000, P.R. China; 3 Department of Pathology, Hebei United University, Tangshan, Hebei 063000, P.R. China; 4 Department of Pathology, Tangshan Worker Hospital, Hebei Medical University, Tangshan, Hebei 063000, P.R. China

**Keywords:** NSCLC, semaphorin-3A, MMP-14, biomarker, prognosis

## Abstract

Semaphorin-3A is a chemorepellent guidance protein that is crucial in regulating the tumor microenvironment. MMP-14, a membrane-anchored matrix metalloproteinase, is closely associated with extracellular matrix (ECM) remodeling and cell migration in the progression of cancer metastasis. In the present study, the correlation between the expression levels of semaphorin-3A and MMP-14, and their subsequent prognostic significance in non-small cell lung cancer (NSCLC), was investigated. The expression of semaphorin-3A and MMP-14 protein levels was analyzed in 94 cases of NSCLC tissues and in 80 cases of normal lung tissues, using immunohistochemistry (IHC). Correlation and survival analysis were used to further investigate their association and prognostic value. The results revealed that the NSCLC tissues exhibited a lower expression of semaphorin-3A and a higher expression of MMP-14 than in the control lung tissues. The downregulation of semaphorin-3A and upregulation of MMP-14 may promote pleural invasion, lymph node metastasis, vascular invasion and proliferating cell nuclear antigen expression. The expression of semaphorin-3A was correlated with the maximum diameter of tumor. There was a negative correlation between the protein expression levels of semaphorin-3A and MMP-14 in NSCLC tissues. Furthermore, we identified that the patients with lower expression of semaphorin-3A and a higher expression of MMP-14 had worse disease prognosis. The data suggest that lower expression of semaphorin-3A and a higher expression of MMP-14 may promote occurrence and development in NSCLC and that the combined detection of semaphorin-3A and MMP-14 protein may be a helpful tool in predicting the prognosis of NSCLC.

## Introduction

Lung cancer ranks among the most common malignant diseases and is currently the leading cause of cancer-related mortality worldwide (1). Lung cancers are divided into two main classes, depending on their histological appearance and presumed cellular origin. Small cell lung cancer (SCLC) is of neuroendocrine origin, while non-small cell lung cancer (NSCLC) is predominantly of epithelial origin. NSCLC accounts for >85% of all lung cancer cases (2). The current treatment methods of NSCLC most commonly include surgery, chemotherapy, radiation therapy and targeted therapy. While early-stage lung cancer may be eminently curable by surgery (3), the majority of lung cancer cases are detected at advanced stages, so combined therapies cannot stand to benefit all patients. The overall 5-year survival rate of lung cancer patients stands at <15%, so it remains a necessity that specific biomarkers and therapeutic targets are explored for the development of novel diagnostic and treatment strategies for the future.

Semaphorins, initially called collapsins, are a large family of secreted, transmembrane and glycosylphosphatidylinositol (GPI)-linked proteins, which were first characterized for their role in axonal guidance in the developing nervous system (4–6). Semaphorins are expressed in numerous tissues where they regulate cell survival, apoptosis, adhesion, directional cell migration, and affect the cytoskeleton, actin filament organization and microtubules (7,8). Based on structural features and amino acid sequence similarity, semaphorins have been divided into eight classes, with classes three to seven representing the vertebrate proteins. Semaphorin-3A is a chemorepellent with multiple guidance functions, including axon pathfinding, cardiac and peripheral vascular patterning and branching morphogenesis (9). Semaphorin-3A signaling is mediated by a complex of the binding receptor neuropilin 1 (NP1) and the signaling receptors plexin A_1_ or A_3_ (10). The action of semaphorin-3A is not limited to the nervous system, as NP1 is expressed on endothelial cells, T cells, keratinocytes and tumor cells. It can inhibit angiogenesis, proliferation of T cells, migration of keratinocytes and tumor cells. In addition, it was recently revealed that semaphorin-3A is involved in the entry of dendritic cells to the lymphatic system. Several studies have demonstrated that overexpression of semaphorin-3A attenuates invasion and matrigel adhesion of tumor cells in certain types of cancer, including prostate and breast (11). Semaphorin-3A has been considered as a potent tumor suppressor in certain malignant neoplasms (12).

Matrix metalloproteinases (MMPs) are the main enzymes involved in the degradation of the extracellular matrix (ECM)and are important in regulating tumor invasion and metastasis (13). Among this family of Zinc-dependent proteolytic enzymes, 28 species have been identified. According to their differences in structure and degradable substrate, MMPs are categorized into four types: Collagenases, gelatinases, matrilysin and membrane-type MMP (14). MMP-14, also known as membrane type-1 MMP, was first discovered in the membrane-type MMP family, at ~3.5 kb, encoding a protein containing 582 amino acids and with a molecular weight of ~66 kda (15). Besides its expression in fibroblasts, smooth muscle and endothelial cells, MMP-14 is also found in the majority of tumor types, including skin, lung, stomach, colon, liver, kidney, breast, bladder and brain, and is one of the most closely linked enzymes, of the MMP family, to the molecular mechanisms of tumor invasion and metastasis (16,17). MMP-14 can degrade many macromolecular ECM components, including type I–III collagen, laminin, fibronectin, vitronectin, cellulose and proteoglycan, by enhancing the hydrolysis effects of the pro-enzymes MMP-2 and MMP-13. MMP-2, in particular, is important in the processes involved in tumor invasion. MMP-14 can affect cell migration in the ECM either through the effects of different intercellular adhesion molecules or by directly providing non-invasive potential cells with the ability to penetrate type I collagen, which is a process that does not depend on the activation of proenzyme MMP-2. MMP-14 increases expression of vascular endothelial growth factor (VEGF), promotes migration of endothelial cells in the ECM and thereby contributes to the formation of new blood vessels. Together, these effects establish an optimal microenvironment to promote tumor invasion and metastasis (17,18).

The present study aimed to identify a correlation between semaphorin-3A and MMP-14 protein expression in NSCLC. Semaphorin-3A and MMP-14 protein expression were first examined in NSCLC and normal lung tissues as the control. Their expression levels were analyzed against clinical characteristics, including pleural invasion, lymph node metastasis, the number of metastatic lymph node, degree of differentiation, vascular invasion and proliferating cell nuclear antigen (PCNA) expression. Finally, the correlation of semaphorin-3A and MMP-14 expression with prognosis was investigated using statistical methods.

## Materials and methods

### Tissue samples and patients

NSCLC tissues (46 cases of lung pulmonary squamous cell carcinoma and 48 cases of pulmonary adenocarcinoma) and normal lung tissues (80 cases) were collected from patients who underwent surgery at the Department of Thoracic Surgery, Tangshan Worker Hospital of Hebei Medical University (Tangshan, China) between January and November 2007. All tumor tissues were histopathologically diagnosed by at least two trained pathologists. Written informed consent was obtained from all patients prior to surgery and the study protocol was approved by the Institutional Review Board for the use of Human Subjects at Tangshan Worker Hospital. The NSCLC group was composed of 60 male and 34 female patients aged 43–75 years (mean, 53.8 years). The control group was composed of 60 male and 20 female patients aged 44–77 years (mean, 53.8 years). None of the patients received pre-operative chemotherapy or radiation therapy. The tissues were immersed in 10% formalin with a pH value of 7.4. Following dehydration in graded ethanol and xylene, the specimens were paraffin-embedded and cut into 4 μm-thick coronal sections.

### HE staining

The sections were stained using hematoxylin/eosin according to the standard procedures and observed under an Olympus BX51 light microscope (Olympus Corporation, Tokyo, Japan).

### Semaphorin-3A and MMP-14 immunohistochemistry

The procedures were processed according to the protocols recommended for the mouse anti-human semaphorin-3A and MMP-14 monoclonal antibodies (Boster Biological Technology Co., Ltd., Wuhan, China). After being deparaffinated and rehydrated, sections were irradiated in 0.1 mol/l sodium citrate buffer (pH 6.0) in a microwave oven (medium/low temperature) for 12 min. Subsequently, the sections were exposed to 3% H_2_O_2_ for 10 min to bleach endogenous peroxidases, followed by rinsing three times in phosphate-buffered saline (PBS) for 10 min. Sections were respectively incubated with a mouse anti-human semaphorin-3A monoclonal antibody (1:100) and a mouse anti-human MMP-14 monoclonal antibody (1:50) for 1 h at 37°C, washed three times in PBS and incubated in a biotinylated goat secondary anti-mouse polyclonal antibody (Boster Biological Technology Co., Ltd.) for 30 min at 37°C. The specificity of the antibodies was examined by omission of the primary antibodies. Following being washed in PBS, the tissues were visualized with 3,3′-diaminobenzidine tetrahydrochloride (DAB) and counterstained with hematoxylin. Finally, the sections were dehydrated in graded ethanol, immersed in xylene and coverslipped.

### Semaphorin-3A and MMP-14 assay

The positive staining of semaphorin-3A and MMP-14 was located in the cytoplasm, and the cells with brown/yellow particles were considered as positive ones. The zone with concentrated positive cells was selected and the number of positive cells in 10 randomly chosen high-power fields (original magnification, ×400) was counted. If the average expression rate of positive tumor cells per high-power field was ≥25%, it was judged as positive, while that <25%, was judged as negative. Finally, the positive rate was calculated.

### Statistical analysis

Statistical analysis was conducted using SPSS for Windows software, version 17.0 (SPSS, Inc., Chicago, IL, USA). The χ^2^ test was used to compare the difference in semaphorin-3A and MMP-14 expression between NSCLC and normal tissues, and between different clinical characteristics of patients in the NSCLC group. Bivariate correlation analysis (Pearson’s product moment coefficient) was used to analyze the correlation between semaphorin-3A and MMP-14 expression in the NSCLC group. A life table was used to calculate survival function and a log-rank test was used for survival analysis. P<0.05 was considered to indicate a statistically significant difference.

## Results

### Expression of semaphorin-3A and MMP-14 in NSCLC tissues and normal lung tissues

**Figure 1:**
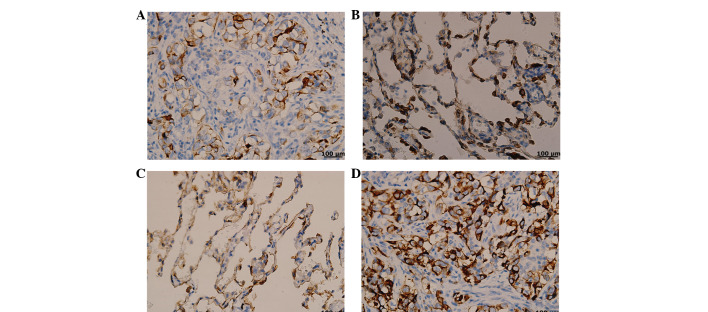
(A) Semaphorin-3A was expressed at low levels in tumor cells and a few brown/yellow particles were observed in the cytoplasm; (B) semaphorin-3A was highly expressed in normal lung cells and a mass of brown/yellow particles was observed in the cytoplasm; (C) MMP-14 was expressed at low levels in normal lung cells and a few brown/yellow particles were observed in the cytoplasm; (D) MMP-14 was highly expressed in the normal lung cells and a mass of brown/yellow particles was observed in the cytoplasm (magnification, ×400).

Semaphorin-3A expression in NSCLC tissues was lower compared with that in the control tissues (Fig. 1A and B), while the MMP-14 expression was higher than that in normal tissues (Fig. 1C and D). In Table I, the data demonstrates that compared with the control group, the NSCLC group had a low positive rate of semaphorin-3A expression (36.17 vs. 75.00%, respectively) and a high positive rate of MMP-14 (75.53 vs. 25.00%, respectively), and the difference was considered to be statistically significant (P<0.05).

### Expression of semaphorin-3A and MMP-14 in patients presenting with different clinical characteristics

In the NSCLC group, the positive rates of semaphorin-3A and MMP-14 expression were relevant to pleural invasion, lymph node metastasis, the number of metastatic lymph nodes, the degree of differentiation, vascular invasion and PCNA expression. In addition, the expression of semaphorin-3A correlated with the maximum diameter of the tumor, while MMP-14 expression revealed no such association (Table II).

### Correlation between the expression of semaphorin-3A and MMP-14 in the NSCLC group

**Figure 2:**
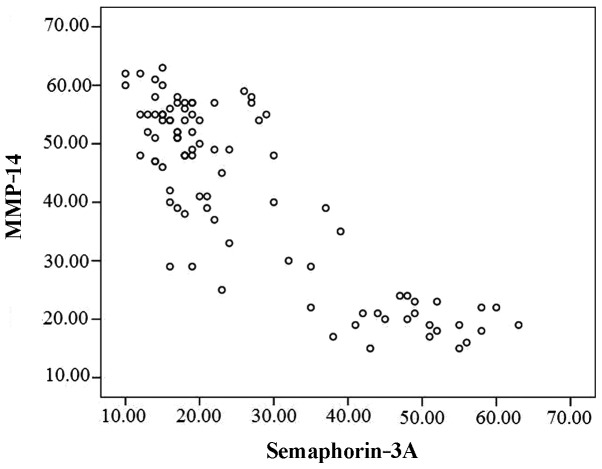
Linear correlation analysis showed that there was a negative correlation between semaphorin-3A and MMP-14 expression.

The linear correlation analysis revealed that semaphorin-3A expression was negatively correlated with MMP-14 expression (r=−0.852, P<0.001; Fig. 2).

### Survival analysis of semaphorin3-A and MMP-14 expression in the NSCLC group

**Figure 3:**
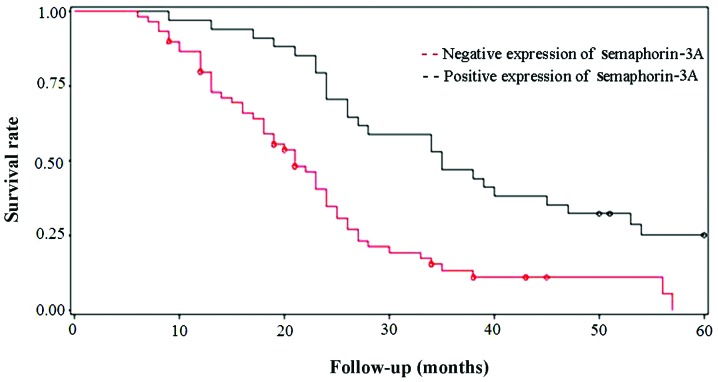
Survival rate of non-small cell lung cancer patients with positive semaphorin-3A expression compared with patients with negative expression. Log-rank test demonstrated that the survival rate of patients with negative semaphorin-3A expression was significantly reduced.

**Figure 4:**
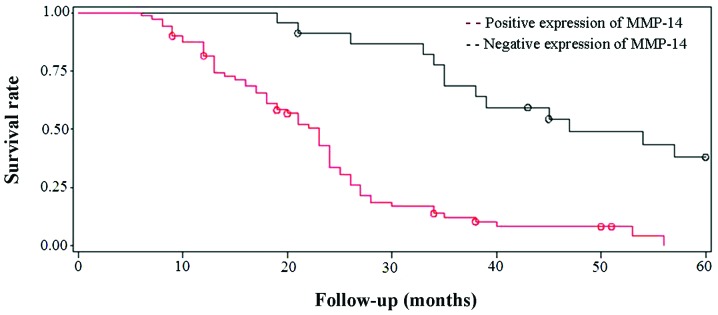
Survival rate of non-small cell lung cancer patients with positive MMP-14 expression compared with patients with negative expression. Log-rank test demonstrated that the survival rate of patients with positive MMP-14 expression was significantly reduced.

The clinical findings of NSCLC patients during long-term follow-up were analyzed and compared with the expression patterns of semaphorin-3A and MMP-14. The follow-up time was from 6 to 60 months (mean, 24.2 months). A life table was used to calculate the survival function of patients with semaphorin-3A and MMP-14 expression, and then the log-rank test was utilized for survival analysis. The results indicated that the protein expression levels of semaphorin-3A and MMP-14 were associated with survival time. Patients with a lower expression of semaphorin-3A and a higher expression of MMP-14 had a worse disease prognosis (Figs. 3 and 4).

## Discussion

Semaphorin-3A is an important protein that belongs to a family of nerve axon guidance factors, which have a crucial physiological role in nervous system development. As the product of a tumor suppressor gene, its role in malignant tumors has attracted much attention recently. Semaphorin-3A has numerous diverse biological functions, including lymphocyte activation, vascular endothelial cell migration, lung and bronchial morphogenesis and promoting tumor cell migration (19). MMPs are a group of proteins with high structural homology which, through a series of proteolytic enzymatic reactions, play a key role in the degradation of the ECM. MMP-14 is important within the MMP family, for its ability to activate other members, including the cell-surface expressed MMP-2, which may be critically involved in accelerating malignant processes, such as tumor metastasis (20). Through the inhibitory control of MMP activation, semaphorins may regulate the breakdown of the ECM components and have thus been implicated as suppressors of tumor metastasis (21).

In the present study, the expression profiles of semaphorin-3A and MMP-14 in NSCLC tissues were examined. The results revealed low level expression of semaphorin-3A and high expression levels of MMP-14. These abnormal expression patterns may have an important role in tumor development and metastasis, and suggest that semaphorin-3A may be a suppressor gene and MMP-14 an oncogene. The experimental results demonstrated that there is a negative correlation between semaphorin-3A and MMP-14 expression in NSCLC, suggesting that these protein levels have negative synergy and promote tumor progression in a collaborative manner. The mechanisms underlying this effect may be explained by the hypothesis that the diverse biological effects of semaphorin-3A are regulated by the NP receptor (22). Since the NP receptor is also the receptor of VEGF isomer, semaphorin-3A may act to limit the combined effects of the VEGF and NP receptor complex, when it is interacting with the NP receptor. As VEGF is a key regulator of blood vessel formation, semaphorin-3A may prevent tumor progression by competitively inhibiting VEGF-induced tumor angiogenesis. As mentioned above, semphorin-3A can induce tumor cell migration by reducing MMP-14 secretion and thus regulate ECM degradation. Recently, it has been hypothesized that MMP-14 also has a role in promoting VEGF secretion and can activate tumor angiogenesis (23). So, it appears the regulatory effects of semaphorin-3A and MMP-14 on cancerous tumors may be associated with VEGF. That is, VEGF is the molecule mediating the synergistic effects of semaphorin-3A and MMP-14 on tumor progression and metastasis. However, its specific mechanisms need to be confirmed by further investigations.

The present study demonstrated that both the expression of semaphorin-3A and MMP-14 in the observation group were closely associated with pleural invasion, lymph node metastasis, the number of metastatic lymph nodes, the degree of differentiation, vascular invasion and expression of PNCA. This result indicated that semaphorin-3A and MMP-14 may be synergistically involved in the processes of tumor invasion, differentiation and vascular dissemination. In addition, the expression of semaphorin-3A was correlated with the maximum diameter of the tumors; the lower the expression, the larger the maximum diameter of the tumor. MMP-14 had a weaker correlation with tumor diameter. Therefore, lower expression of semaphorin-3A exhibited a more evident promoting effect on tumor growth. PNCA, as an important indicator of tumor proliferation index, is more strongly associated with semaphorin-3A and MMP-14. The abnormal expression levels of semaphorin-3A and MMP-14 appear to promote tumor cell proliferation, which provides direct evidence for its damaging impact on tumor progression and disease prognosis. Survival analysis identified that the patients with low expression of semaphorin-3A and high expression of MMP-14 had a worse prognosis. Therefore, the combined detection of semaphorin-3A and MMP-14 postoperatively is significantly valuable on the judgment of prognosis of NSCLC.

The present study demonstrated that low expression of semaphorin-3A and high expression of MMP-14 in NSCLC may promote tumor progression, possibly due to negative synergy, and the combined detection of semaphorin-3A and MMP-14 postoperatively was valuable in the judgment of prognosis. The upregulation of semaphorin-3A and downregulation of MMP-14 may provide a useful strategy for future NSCLC inhibitory therapies.

## Figures and Tables

**Table I:** Comparison of semaphorin-3A and MMP-14 expression between the NSCLC group and control group.

		Semaphorin-3A				MMP-14			
							
Variable	n	+ (%)	− (%)	χ^2^	P-value	+ (%)	− (%)	χ^2^	P-value
NSCLC group	94	34 (36.17)	60 (63.83)	26.2349	<0.0001	71 (75.53)	23 (24.47)	44.2363	<0.0001
Control group	80	60 (75.00)	20 (25.00)			20 (25.00)	60 (75.00)		

MMP, matrix metalloproteinase; NSCLC, non-small cell lung cancer.

**Table II:** Analysis of semaphorin-3A and MMP-14 expression in the NSCLC group.

		Semaphorin-3A				MMP-14			
							
Variable	n	+ (%)	− (%)	χ^2^	P-value	+ (%)	− (%)	χ^2^	P-value
Pleural invasion				5.6360	0.0176			5.3965	0.0202
No	54	25 (46.30)	29 (53.70)			36 (66.67)	18 (33.33)		
Yes	40	9 (22.50)	31 (77.50)			35 (87.50)	5 (12.50)		
Lymph node metastasis				14.3348	0.0002			8.5288	0.0035
No	37	22 (59.46)	15 (40.54)			22 (59.46)	15 (40.54)		
Yes	57	12 (21.05)	45 (78.95)			49 (85.96)	8 (14.04)		
Number of metastatic lymph nodes				7.2598	0.0071			7.5552	0.0060
<4	64	29 (45.31)	35 (54.69)			43 (67.19)	21 (32.81)		
≥4	30	5 (16.67)	25 (83.33)			28 (93.33)	2 (6.67)		
Expression of PCNA, %				23.4801	<0.0001			5.7913	0.0161
<25	49	29 (59.18)	20 (40.82)			32 (65.31)	17 (34.69)		
≥25	45	5 (11.11)	40 (88.89)			39 (86.67)	6 (13.33)		
Degree of differentiation				8.7037	0.0032			9.4801	0.0021
Well and moderately	56	27 (48.21)	29 (51.79)			36 (64.29)	20 (35.71)		
Poorly	38	7 (18.42)	31 (81.58)			35 (92.11)	3 (7.89)		
Vascular invasion				5.7928	0.0161			6.4769	0.0109
No	66	29 (43.94)	37 (56.06)			45 (68.18)	21 (31.82)		
Yes	28	5 (17.86)	23 (82.14)			26 (92.86)	2 (8.70)		
Maximum diameter of tumor, cm				15.2753	<0.0001			0.4966	0.4810
<5	44	25 (56.82)	19 (43.18)			31 (73.81)	11 (26.19)		
≥5	50	9 (18.00)	41 (82.00)			40 (80.00)	10 (20.00)		

Expression of semaphorin-3A and MMP-14 in patients presenting with different clinical characteristics. MMP, matrix metalloproteinase; NSCLC, non-small cell lung cancer; PCNA, proliferating cell nuclear antigen.
